# Extracellular Vesicles Derived From Regeneration Associated Cells Preserve Heart Function After Ischemia-Induced Injury

**DOI:** 10.3389/fcvm.2021.754254

**Published:** 2021-10-20

**Authors:** Amankeldi A. Salybekov, Ainur Salybekova, Yin Sheng, Yoshiko Shinozaki, Keiko Yokoyama, Shuzo Kobayashi, Takayuki Asahara

**Affiliations:** ^1^Kidney Disease and Transplant Center, Shonan Kamakura General Hospital, Kamakura, Japan; ^2^Shonan Research Institute of Innovative Medicine, Shonan Kamakura General Hospital, Kamakura, Japan; ^3^Division of Regenerative Medicine, Department of Center for Clinical and Translational Science, Shonan Kamakura General Hospital, Kamakura, Japan; ^4^Department of Advanced Medicine Science, Tokai University School of Medicine, Isehara, Japan; ^5^Teaching and Research Support Core Center, Tokai University School of Medicine, Isehara, Japan

**Keywords:** extracellular vesicles, regeneration-associated cells, myocardial ischemia-reperfusion injury, miR, angiogenesis

## Abstract

Under vasculogenic conditioning, pro-inflammatory cell subsets of peripheral blood mononuclear cells (PBMCs) shift their phenotype to pro-regenerative cells such as vasculogenic endothelial progenitor cells, M2 macrophages, and regulatory T cells, collectively designated as regeneration-associated cells (RACs). In this study, we evaluated the therapeutic efficacy of RAC-derived extracellular vesicles (RACev) compared to mesenchymal stem cell-derived EVs (MSCev) in the context of myocardial ischemia reperfusion injury (M-IRI). Human PBMCs were cultured with defined growth factors for seven days to harvest RACs. RACev and MSCev were isolated via serial centrifugation and ultracentrifugation. EV quantity and size were characterized by nanoparticle tracking analysis. *In vitro*, RACev markedly enhanced the viability, and proliferation of human umbilical vein endothelial cells in a dose-dependent manner compared to MSCev. Notably, systemic injection of RACev improved cardiac functions at 4 weeks, such as fractional shortening, and protection from mitral regurgitation than the MSCev-treated group. Histologically, the RACev-transplanted group showed less interstitial fibrosis and enhanced capillary densities compared to the MSCev group. These beneficial effects were coupled with significant expression of angiogenesis, anti-fibrosis, anti-inflammatory, and cardiomyogenesis-related miRs in RACev, while modestly in MSCev. *In vivo* bioluminescence analysis showed preferential accumulation of RACev in the IR-injured myocardium, while MSCev accumulation was limited. Immune phenotyping analysis confirmed the immunomodulatory effect of MSCev and RACev. Overall, repetitive systemic transplantation of RACev is superior to MSCev in terms of cardiac function enhancements via crucial angiogenesis, anti-fibrosis, anti-inflammation miR delivery to the ischemic tissue.

## Introduction

Extracellular vesicles (EVs), as messengers, maintain intercellular communication by delivering various types of proteins, lipids, and genetic materials such as DNA, mRNA, and microRNAs (miRs) to recipient cells (1). EVs are highly heterogeneous in terms of their size, origin, morphology, function, and genetic material carried (1). As EV composition/contents are different under physiological and pathological conditions, EVs are widely used as disease biomarkers or treatment agents based on their unique features (2–4).

Preclinical studies have demonstrated that EVs isolated from endothelial progenitor cells (EPCs), cardiac progenitor cells, and mesenchymal stem cells (MSCs) have strong therapeutic potential compared to their parental cells (5–8). In previous study by Lai et al. showed that MSC-derived exosomes or extracellular vesicles (MSCexo/ev) reduced the infarct size in a mouse model of myocardial ischemia/reperfusion injury (M-IRI) (9). MSCev are also known to exert cardioprotective effects by delivering anti-inflammatory and anti-apoptosis miRs and proteins, resulting in preserved cardiac function after ischemic injury (10, 11).

Previously, we have shown that under vasculogenic conditioning, pro-inflammatory cell subsets of peripheral blood mononuclear cells (PBMCs) such as type 1 macrophages (M1Φ), T cells, and EPCs beneficially shifted their phenotype to pro-regenerative cells such as vasculogenic EPCs, M2 macrophages, and regulatory T cells, which are collectively designated as regeneration-associated cells (RACs) (12, 13). Furthermore, systemic transplantation of a small number of RACs (1 ^*^ 10^5^) significantly improved cardiac function by increasing microvascular density (CD31), strong anti-inflammatory action (type 2 macrophage (M2Φ) and regulatory T cells (Treg) infiltration), and initiation of cardiomyogenesis in a rat myocardial infarction model (12).

In this study, we attempted to elucidate the therapeutic efficacy of RAC-derived EVs (RACev) in the context of myocardial ischemia reperfusion injury (M-IRI).

## Materials and Methods

### RAC and MSC Culture

As indicated by the Tokai University School of Medicine Institutional Review Board, human PBMCs were isolated from healthy human volunteers after obtaining written informed consent. Peripheral blood (30–50 mL) was drawn using a heparinized syringe. Briefly, cells were cultured with five growth factor-supplemented culture medium, Stem Line II (Sigma-Aldrich, St. Louis, MO), with 100 ng/mL recombinant human (rh) stem cell factor (SCF), 100 ng/mL Flt-3 ligand (FL3L), 20 ng/mL thrombopoietin (TPO), 50 ng/mL VEGF, and 20 ng/mL interleukin-6 (IL-6) (all from Peprotech, Inc. (Rocky Hill, NJ, USA), and penicillin/streptomycin (100 U/100 μg/mL) (Gibco). PBMCs at a density of 10 ^*^ 10^7^ cells were cultured for seven days on a 10-cm dish (Sumitomo Bakelite Co. Tokyo. Japan) in a 37°C incubator with a humidified atmosphere of 5% CO_2_, as described earlier (12, 14). Bone marrow-derived MSCs (Lonza, Japan) passage 3–5 were used for all *in vitro* and *in vivo* studies.

### Extracellular Vesicles Isolation and Quantification Labeling

Immediately after harvesting RACs and MSCs, the cells were seeded in X-*vivo* 15 (Lonza, for RACs) supplemented with 5% exosome-depleted FBS (Gibco) and in DMEM supplemented with 5% FBS (Gibco, for MSCs) for 48 h. Conditioned culture medium (CCM) was carefully collected, and the cells were removed by centrifugation at 300 × *g* at 4°C for 10 min. Then, larger apoptotic bodies and microvesicles were removed by centrifugation at 2,000 × *g* for 20 min at 12°C, followed by filtration through a 0.2 μm filter (Millipore, Merck) to remove particles larger than 200 nm. The filtered CCM was transferred to new ultracentrifuge tubes (Ultra clear Beckman cat# 344059, rotor type is Ti40SW, Beckman Coulter), which were then sealed and ultracentrifuged at 174 000 × *g* for 110 min at 4°C to pellet the EVs. Then, EVs number and size was measured using nanoparticle tracking analysis with NanoSight NS500® (Malvern Panalytical, UK) according to manufacturer manual. Morphological characteristics of extracellular vesicles was verified common transmission electron microscopy method (JEM-1400, Jeol. Co.Ltd, Japan).

### Flow Cytometry Analysis of EVs and Immune Cells

All EV flow cytometry analysis experiments were performed using a 13-color, 3-laser DxFlex Flow Cytometer, equipped with 405 nm, 488 nm, and 638 nm lasers (Beckman Coulter, Brea, CA). For analyzing small EVs, the configuration was modified for violet side scatter (VSSC) detection. Briefly, the 405/10 VSSC filter was moved to the V450 channel in the wavelength-division multiplexing (WDM), and the detector configuration was updated using CytExpert software to assign the VSSC channel within the WDM. Before data acquisition, cleaning was performed using with FlowClean and water passed through a 10 μm filter to reduce the debris and background noise. For EV characterization, fluorophore-conjugated antibodies against CD9 (PE, #312106), CD63 (PE, #353004), Alix (Alexa 594, #634504), and Hsp-70 (Alexa 488, #648003) were used (all from BioLegend) according to the manufacturer's instructions. Latex beads of different sizes (100 nm and 200 nm, Beckman Coulter) were used for comparison with EVs and to adjust the flow cytometry acquisition voltages.

The weight of spleen was evaluated before cell isolation. Peripheral blood and spleen-derived mononuclear cells were isolated by density gradient centrifugation using Lymphocyte Separation Solution (d = 1.077; Nakalai Tesque, Kyoto, Japan), as previously reported (12–14). Isolated PBMC and spleen MNCs incubated with FcR blocking (Miltenyi Biotec, Germany) to reduce non-specific antibody bindings. Briefly, cell surface markers staining was carried out using CD3-FITC (cat#401606), CD4-PE/Cy7 (cat#400126), CD8a-AlexaFlour 647 (cat#400130, CD11b/c-PerCP/Cy5.5 (cat#400258), CD161-PE (cat# 205604) (all antibodies from Biolegend CA, USA) at room temperature for 20 min, following which cells were washed twice with 2 mmol/L EDTA/0.2% BSA/PBS buffer. The data were acquired on a FACS Verse (BD Biosciences) and analyzed with FlowJo software, version 10.7.

### Cell Proliferation and EV Internalization Assays

For the cell proliferation assay, human umbilical vein endothelial cells (HUVECs) (Lonza, Japan) at passage 3–4 were used. Briefly, HUVECs were harvested and incubated with Cytopainter dye (Cat# ab176735) at 37°C for 10–30 min. The working dye solution was removed by two rounds of centrifugation and the labeled cells were seeded with the control group (HUVEC only). After filtration of CCM through a 0.22μm filter (Millipore Merck), 2 μM CM-DiI dye (C7000, Thermo Fisher) was added to the CCM and left in incubator for 30 min prior to ultracentrifugation at 174 000 × g for 110 min at 4 °C. The labeled EVs were enriched at the bottom of the tube and were washed twice by ultracentrifugation in 1X phosphate-buffered saline (PBS) (15). Then, RACev and MSCev added to labeled HUVEC in a dose-dependent manner (EVs-derived from 2.5 or 5 ^*^ 10^5^ either RACs or MSCs). After 2 days, the cells were isolated and analyzed using flow cytometry (BD Biosciences, FACS Verse).

For the invasion assay, HUVECs were stained with Hoechst (Cat #33342) dye according to the manufacturer's instructions. Finally, pure labeled EVs were mixed with 3 × 10^5^ HUVECs and analyzed under a confocal microscope (Zeiss LSM 880, Germany).

### Myocardial Ischemia-Reperfusion Injury

All animal experiments were performed in accordance with the Tokai University School of Medicine Animal Care and Use Committee (approval # I-20056) and were based on the Guide for the Care and Use of Laboratory Animals (National Research Council, Japan). Male Lewis rats (6–10 weeks of age) weighing 150–250 g, were purchased from Charles River Laboratories (Yokohama, Japan) through Oriental Yeast Co. Ltd. (Tokyo, Japan). Animals were maintained under standard conditions (20 ± 2°C, relative humidity 50–60%, 12 h/12 h light/dark cycles), and were monitored daily by the Animal Support Center for Medical Research and Education at Tokai University School of Medicine. The animals had *ad libitum* access to water and food.

For the M-IRI model, the animals were anesthetized with 3–4% sevoflurane (Maruishi Pharmaceutical Co., Ltd. Japan), orally intubated, and respired using a rodent ventilator at 15 mL/kg, 65–70 times/min (Harvard Apparatus, USA) (16). After left-sided thoracotomy, myocardial ischemia reperfusion injury was induced by temporary occlusion of the left anterior descending artery for 30 min and subsequent reperfusion. Immediately after thorax closure, animals underwent transplantation of PBS-diluted RACev and MSCev (EVs derived from 5 ^*^ 10^5^ either RACs or MSCs) via the tail vein using a 24G angiocatheter (Terumo, Japan) as described previously (12). After the completion of each experiment, the animals were sacrificed by an overdose of sevoflurane 5%; after confirming euthanasia, vital organs such as the lung and aorta were harvested.

### Echocardiography Assessment

Echocardiography (Aloka Co., Ltd., Tokyo, Japan) with a 3.5-MHz probe was used to determine the left ventricular end-diastolic dimension (LVDD), end-systolic dimension (LVDS), severity of mitral regurgitation (MR), the height of the E-wave and A-wave, and the size of the left atrium and ascending aorta. These measurements were repeated before M-IRI induction (baseline) and then weekly following M-IRI injury for 4 weeks. The severity of mitral regurgitation was defined by the size of the regurgitation flow reaching the left atrial wall (severe, score 3), 2/3 of the left atrium (moderate, score 2), and 1/3 of the left atrium (mild or trace, score 1). Fractional shortening (%FS) was defined as (LVDD-LVDS)/LVDD × 100 (12, 16).

### Immunohistochemistry Analysis

The heart was perfused with heparinized PBS (1,000 U/500 mL) and fixed overnight with 4% paraformaldehyde (PFA, #163-20145, Fujifilm. Co. Ltd.), embedded in paraffin, and cut into 3- to 4-mm-thick sections. The sections were stained with picrosirius red (Muto Co., Ltd. Japan) to determine the size of interstitial fibrosis, as described previously (11). The immunohistochemistry data were evaluated from 3 to 5 high-power fields per section in a blinded manner using Image J (version 1.51, National Institutes of Health). The sections were blocked in an avidin and streptavidin complex and incubated with isolectin B4-FITC (1:100) (Invitrogen, CA, USA) to evaluate the infarcted tissue microvascular density (MVD) (12). To discriminate auto-fluorescence, spectral analysis of stained tissue sections was performed using a Carl Zeiss LSM880 meta confocal microscope (Oberkochen, Germany).

### EV Tracking

The CCM was stained with DiI, as mentioned above (in section Cell Proliferation and EV Internalization Assays). Briefly, labeled MSCev and RACev, and the control solution (saline) were injected via the tail vein using a 24G angiocatheter (Terumo) immediately after releasing the ligated left anterior descending artery. After 3.5 h of injection, the animals were sacrificed and the heart was excised for further EV tracking analysis as described elsewhere (15, 17). Briefly, in *vivo* imaging system (IVIS) Lumina III (Perkin Elmer, USA) was used to analyze EV entrapment at the site of injury. DiI dye excitation and emission wavelength filters were set at 520 nm and 570 nm, respectively (15). Frozen heart sections of 6 μm thickness were then analyzed under a LSM 880 confocal microscope (Carl Zeiss, Germany).

### EV-Derived Total RNA Isolation, Library Preparation, and Sequencing

According to the manufacturer's protocol, CCM-derived small and large RNAs were fractionated using the miRNeasy mini kit (Qiagen, USA) and the RNeasy MinElute Cleanup Kit (Qiagen). Sequencing libraries were constructed according to the manufacturer' s protocols using the QIAseq™ miRNA Library Kit (Cat#331505, Qiagen, Hilden, Germany). Library quality was assessed with an Agilent Bioanalyzer using a High Sensitivity DNA chip (Agilent Technologies, Santa Clara, CA, USA). The pooled libraries were sequenced using NextSeq 500 (Illumina, Inc., San Diego, CA, USA) in 76-base-pair (bp) single-end reads.

### Bioinformatics Analysis of Differentially Expressed miRs

The QIAseq miRNA library kit adopts a unique molecular index (UMI) system, enabling unbiased and accurate quantification of mature miRs. Original FASTQ files generated using NextSeq were uploaded to the Qiagen GeneGlobe Data Analysis Center (https://geneglobe.qiagen.com) and aligned to miRBase v21 (http://www.mirbase.org). All reads assigned to particular miRs were counted, and the associated UMIs were aggregated to count unique molecules. A matrix of the miR UMI counts was subjected to downstream analyses using StrandNGS 3.4, software (Agilent Technologies, Santa Clara, CA). The UMI counts were quantified using the trimmed mean M-value (TMM) method (18). To determine the target genes of differentially expressed miRs, we integrated all the information on PITA miRBase version 18, microRNA.org miRBase version 18, and TargetScan version 6.0. Next, to determine the biological function of the target genes, Gene Ontology (GO) analysis and pathway statistical analysis were performed. Pathway statistical analysis to determine the pathways considering the number of genes in the pathway and the number of target genes was performed on the pathway collection of the Wiki Pathways database (19) using the PathVisio tool (20).

### Statistical Analysis

All values are shown as mean ± SEM. Mann-Whitney U and Kruskal Wallis tests were utilized for two and three non-parametric groups, followed by Dunn's multiple comparison tests. Also, one-way-ANNOVA was used for three or more parametric groups, followed by Dunn's or Tukey's multiple comparison tests. For multiple comparisons between groups at different time points, 2-way ANOVA was applied, followed by Tukey's *post-hoc* test. Differentially expressed miRs were determined using a threshold of absolute values of fold change ≥2 and FDR <0.05 (moderated *t*-test followed by Benjamini-Hochberg multiple testing correction). All statistical analyses were performed using GraphPad Prism 9.1 (GraphPad Prism Software Inc., San Diego, CA, USA). Statistical significance was set at *P* < 0.05.

## Results

### Characterization of EVs

**Figure 1:**
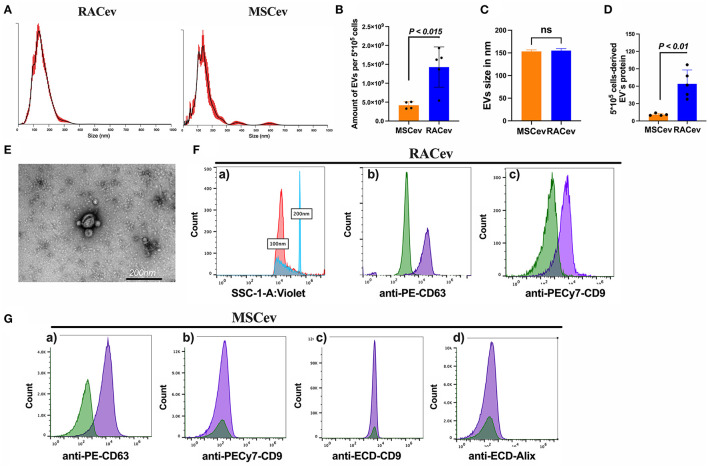
Characterization of extracellular vesicles. **(A–C)** Nano tracking particle analysis of EV characteristics such as size and quantity. **(D)** Representative transmission-electron microscopy figures showing the lipid bilayer structure of EVs. Distinct EV biomarkers expression profiles such as CD9, CD63, and Alix of **(E,F)** RACev and **(G)** MSCev. Statistical significance was determined using Mann-Whitney U test. The results are presented as mean ± SEM (*n* = 4–7 per group).

The number and size of EVs derived from RACs and MSCs measured by nanoparticle analyzing machine (Figures 1A–C). RACs secrete more EVs (1.62 x 10^9^ vs. 4.18 x 10^8^, *P* < 0.015) compared to MSCs per 5^*^10^5^ cells (Figure 1B). The average diameter of RACev was slightly larger (155 ± 1.7 nm) than MSCev (153.3 ± 2 nm) (Figure 1C). Protein content evaluation assay demonstrated that RACev protein level is 6-fold higher than MSCev (11 ± 1 vs. 64 ± 10, *P* < 0.01). Transmission electron microscopy revealed a bi-layered membrane structure (Figure 1D), and flow cytometry confirmed the presence of EV-specific markers (CD9, CD63, and Alix) (Figures 1E,F). Interestingly, not all classical EV markers are expressed equally; for example, RACev highly expressed CD9, whereas MSCev showed modest expression (Figure 1G). Here, we showed that flow cytometry analysis could be utilized as a size evaluation and EV quantification tool using beads of different sizes and appropriate dilutions of EVs (Figure 1Fa).

Together, the isolated RACev and MSCev expressed EV-specific markers, and their size range and TEM findings complied with the MISEV 2018 guidelines (21).

### RACev Showed Enhanced Proliferative and Anti-apoptotic Effects on HUVECs in a Dose-Dependent Manner

**Figure 2:**
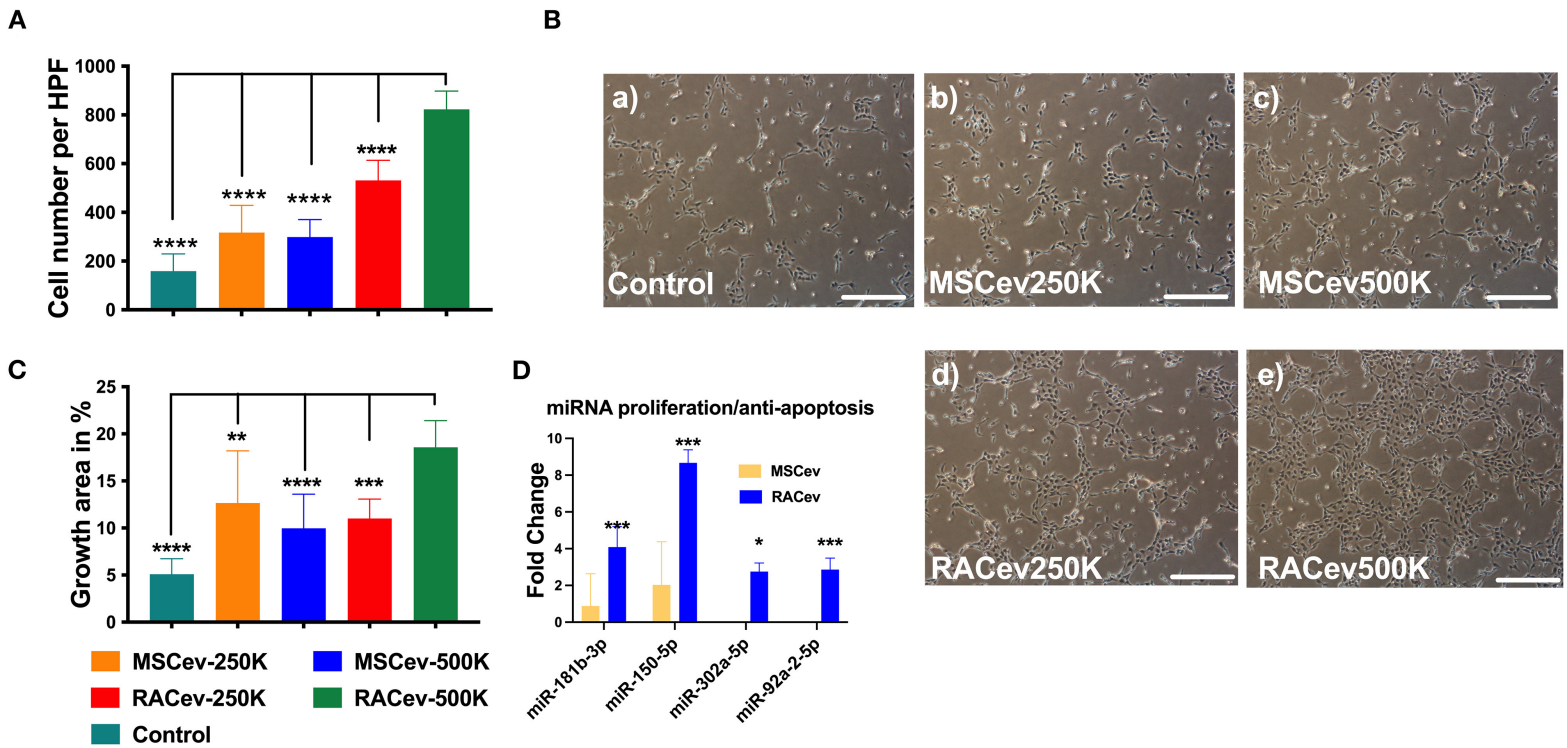
RACev enhanced endothelial cell proliferation in a dose-dependent manner. **(A,B)** 5 *10^5^ RACs-derived EVs (500K) increased the cell numbers and **(B,C)** growth area in culture dishes. **(D)** The miRs responsible for proliferation and anti-apoptosis were markedly upregulated in RACev vs. MSCev. * *P* < 0.05; ** *P* < 0.01; *** *P* < 0.001; **** *P* < 0.0001 for RACev-500K vs. other groups. Statistical significance was determined using one way ANOVA followed by Tukey's multiple comparison test. The results are presented as mean ± SEM (*n* = 7–10 per group).

When HUVECs were co-cultured with different concentrations of RAC-derived EV and MSC-derived EV (2.5 ^*^ 10^5^ and 5 ^*^ 10^5^), we observed a highly proliferative effect of RACev in a dose-dependent manner compared to MSCev (MSCev-250K vs. RACev-250K, *P* < 0.001 and MSCev-500K vs. RACev-500K, *P* < 0.0001) (Figures 2A,B). Of note, MSCev increased proliferation effect and growth area of HUVECs compared to control groups (Figures 2A–C). However, growth area of HUVECs was significantly enhanced in the RACev co-culture group compared to that in the MSCev co-culture group (Control vs. RACev-500K, *P* < 0.0001 and MSCev-500K vs. RACev-500K, *P* < 0.0001) (Figures 2B,C). Cell cycle analysis confirmed that most of the HUVECs co-cultured with RACev at 250 K and 500 K concentrations entered the S phase and showed increased cell division by up to seven-cell generations (Supplementary Figure 1). We then analyzed cell cycle/proliferation-related miR expressional profiles in the RACev vs. MSCev and found that a key miR responsible for anti-apoptotic effects, cell division, and migration was significantly upregulated in RACev (Figure 2D). Our *in vitro* results showed that RACev increased the cell division cycle by delivering crucial anti-apoptotic and cell division miRs compared to MSCev.

### RACev Transplantation Preserved Heart Function

**Figure 3:**
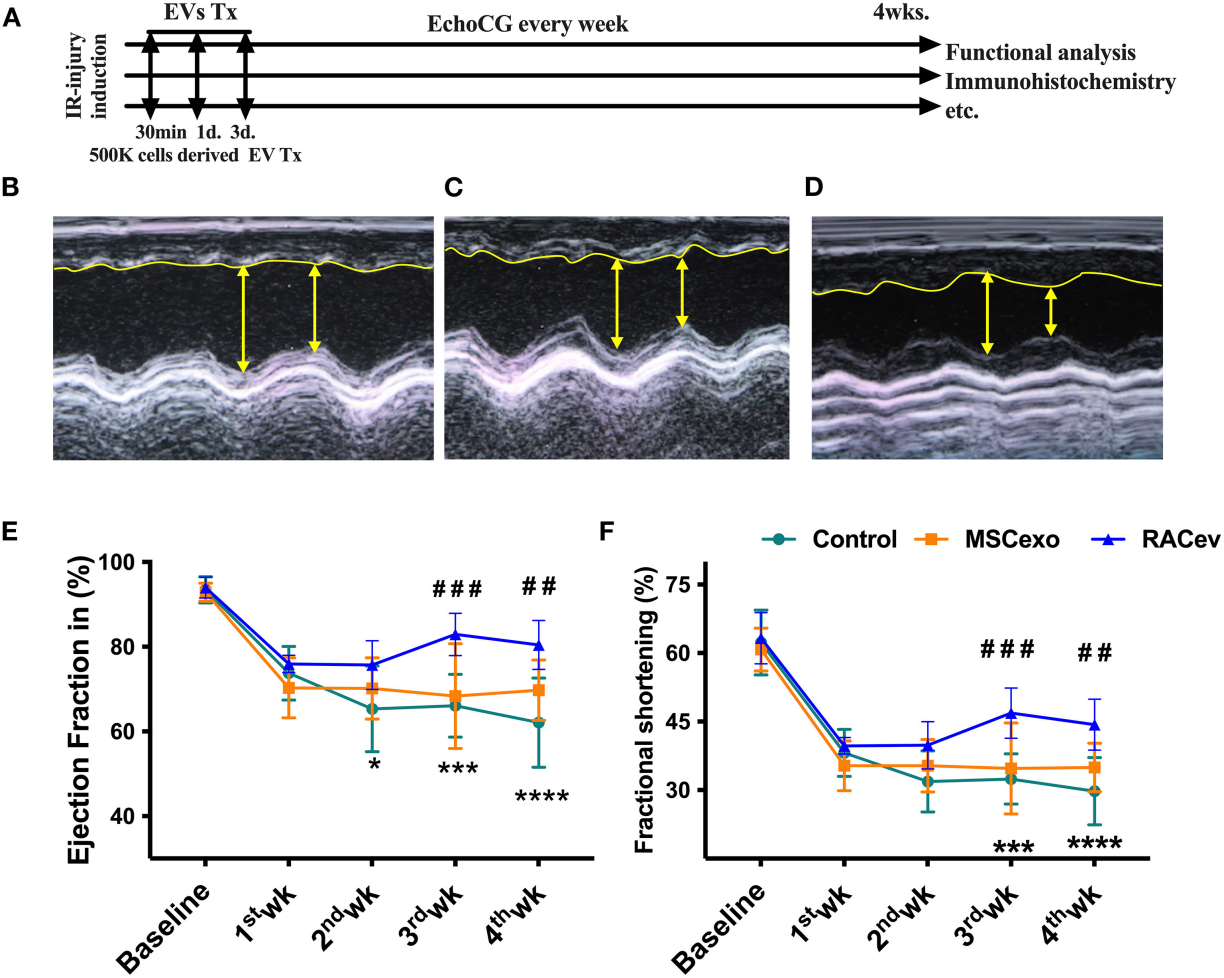
RACev transplantation improved cardiac function. **(A)** Schematic of *in vivo* experiment design. **(B–D)** Representative echocardiographic images of the Control, MSCev, and RACev groups. **(E,F)** The RACev transplanted group showed preserved ejection fraction and fractional shortening at 4 weeks. *P < 0.05; ***P < 0.001; ****P < 0.0001 vs. the Control group; ^*##*^*P* < 0.01; ^*###*^*P* < 0.001 vs. the MSCev group. Scale bar = 100 μm Statistical significance was determined using 2-way ANOVA followed by Tukey's multiple comparison post-hoc test. The results are presented as mean ± SEM. (*n* = 8–10 per group). EchoCG, echocardiography; EV, extracellular vesicles; M-IRI, myocardial ischemic reperfusion injury; Tx, transplantation; 500K is 5 x 10^5^ either RACs or MSCs-derived EVs.

**Figure 4:**
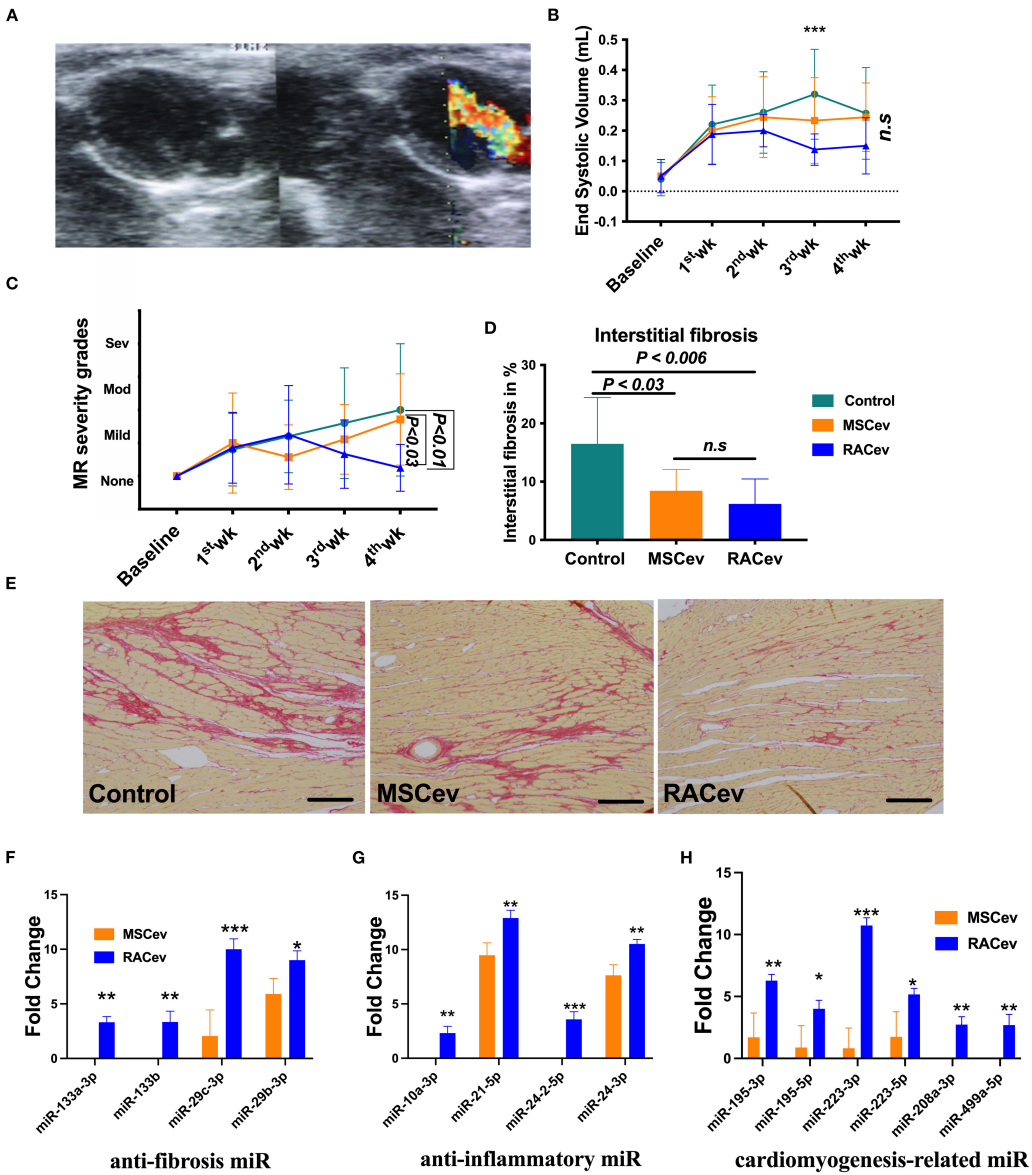
RACev transplantation preserved mitral regurgitation (MR) and interstitial fibrosis. **(A)** Representative mitral regurgitation echocardiographic 2D image. **(B)** At 4 weeks, mitral regurgitation incidences were higher in the Control and MSCev-treated groups than the RACev group. **(C)** Mitral regurgitation caused end-systolic volume increase in the control and MSCev-treated groups but not in the RACev group. **(D, E)** Interstitial fibrosis accumulation was greater in the control group than in the MSCev and RACev groups. **(F)** Anti-fibrosis miRs were significantly upregulated in RACev vs. MSCev. Furthermore, miR-133a-3p and miR-133b are expressed in RACev but not MSCev **(G)** Previously defined anti-inflammation related miRs such as miR10a-3p and miR-24-2-5p were expressed only in RACev group (RACev vs. MSCev). **(H)** Cardiomyogenesis-related miR significantly upregulated in RACev vs. MSCev, and miR-208a-3p and miR499a-5p detected in RACev group but not in MSCev. Scale bar = 100μm *P < 0.05; **P < 0.01; ***P < 0.001 vs. the MSCev group. Statistical significance was determined using 2-way ANOVA (for MR and ESV) or one-way ANNOVA (for interstitial fibrosis) followed by Dunn's or Tukey‘s multiple comparison post-*hoc* tests. The results are presented as mean ± SEM (*n* = 8–10 per group).

Based on the previous preclinical data (12) and *in vitro* data, we adjusted the treatment dose to 5 ^*^ 10^5^ cells derived EVs either RAC or MSC, and injected them repetitively (30 min, day 1, and day 3 after IRI) via tail vein (Figure 3A). Echocardiography analysis at 4 weeks showed that the ejection fraction was significantly improved in the RACev transplanted group compared to the MSCev and control groups at 4 weeks (*P* < 0.006, RACev vs. MSCev; *P* < 0.0001, RACev vs. Control) (Figures 3B–E). The fractional shortening parameter was similarly improved in the RACev group compared to the MSCev and control groups (*P* < 0.005, RACev vs. MSCev; *P* < 0.0002, RACev vs. Control at 4 weeks) (Figure 3F). After myocardial IRI, mitral valve insufficiency was observed in most animals in the D-flow mode, and this tendency continued in the MSCev- and saline-treated groups; in contrast, RACev transplantation protected the rats from mitral regurgitation at 4 weeks (*P* < 0.03, RACev vs. MSCev; *P* < 0.01, RACev vs. Control) (Figures 4A,C). Consequently, MR caused an end-systolic left ventricle volume increase in the MSCev-treated and Control groups but not in the RACev-treated group (Figure 4B). Then, we evaluated the interstitial fibrosis composition at 4 weeks using picrosirius red staining. As shown in Figures 4D,E, the highest interstitial fibrotic structure content was detected in the control group. RACev and MSCev-treated groups significantly reduced interstitial myocardial fibrosis compared to control group (*P* < 0.006, RACev vs. Control; *P* < 0.03, MSCev vs. Control). This anti-fibrotic effect of RACev may be coupled with anti-inflammation and anti-fibrotic miR delivery to the ischemic tissue which was beneficially preserved from excessive inflammation and scar formation (Figures 4F,G). Interestingly, cardiomyogenesis-related miRs were significantly upregulated in RACev vs. MSCev (Figure 4H), suggesting cardiomyogenic properties of RACev. Taken together, repetitive RACev transplantation significantly augmented heart function parameters via reduced interstitial fibrosis accumulation, anti-inflammation, and cardiomyogenesis-related miR delivery.

### RACev Increased Capillary Densities

**Figure 5:**
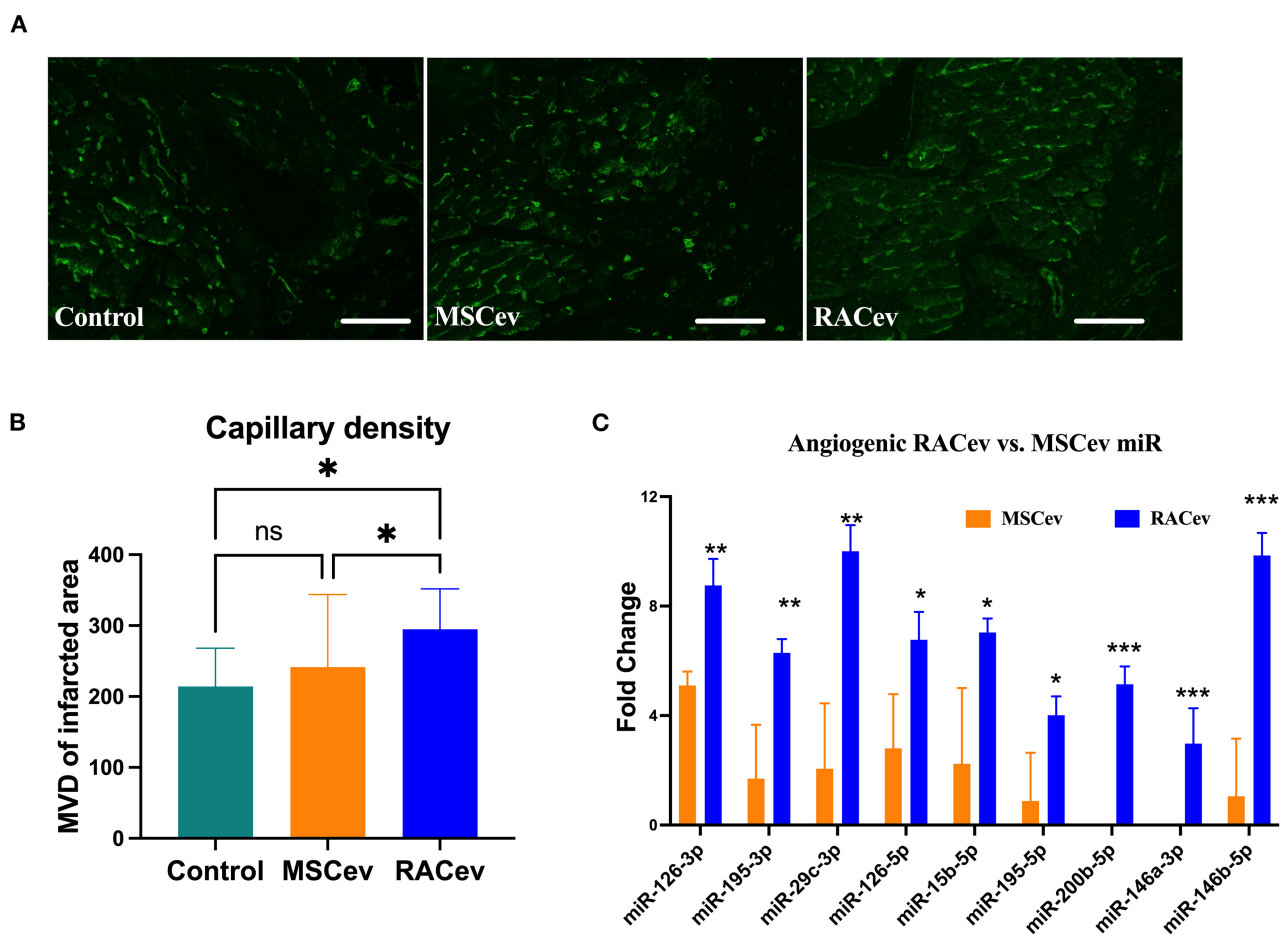
RACev derived angiomiRs enhanced angiogenesis in infarcted tissues. **(A,B)** Microvascular density was enhanced in myocardial infarcted tissue in RACev transplanted group. Cardiac capillaries were stained with FITC conjugated Isolectin B4 (green) **(C)** Mechanistically, enhanced capillary formation in injured myocardium may couple with well-known angiomiRs (miR-126-3p and 5p, miR-195-5p, etc.) delivery by RACev. Scale bar = 50μm. *P < 0.05; **P < 0.01; ***P < 0.001 vs. MSCev group; Statistical significance was determined using Kruskal-Wallis followed by Dunn's multiple comparisons post-*hoc* test. Results are presented as the mean ± SEM (*n* = 8–10 per group).

Early angiogenesis initiation is crucial for the regeneration of ischemic injured organs. Representative Figures 5A,B show that the microvascular densities of the infarcted myocardium were beneficially enhanced in the RACev transplanted group compared to the MSCev and control groups, indicating the strong angiogenic properties of RACev. Moreover, transcriptome analysis showed that the key pro-angiogenic miRs responsible for angiogenesis, called angiomiRs, were more highly upregulated in RACev than in MSCev (Figure 5C).

### Evidence of Selective RACev Accumulation at the Site of Injury

**Figure 6:**
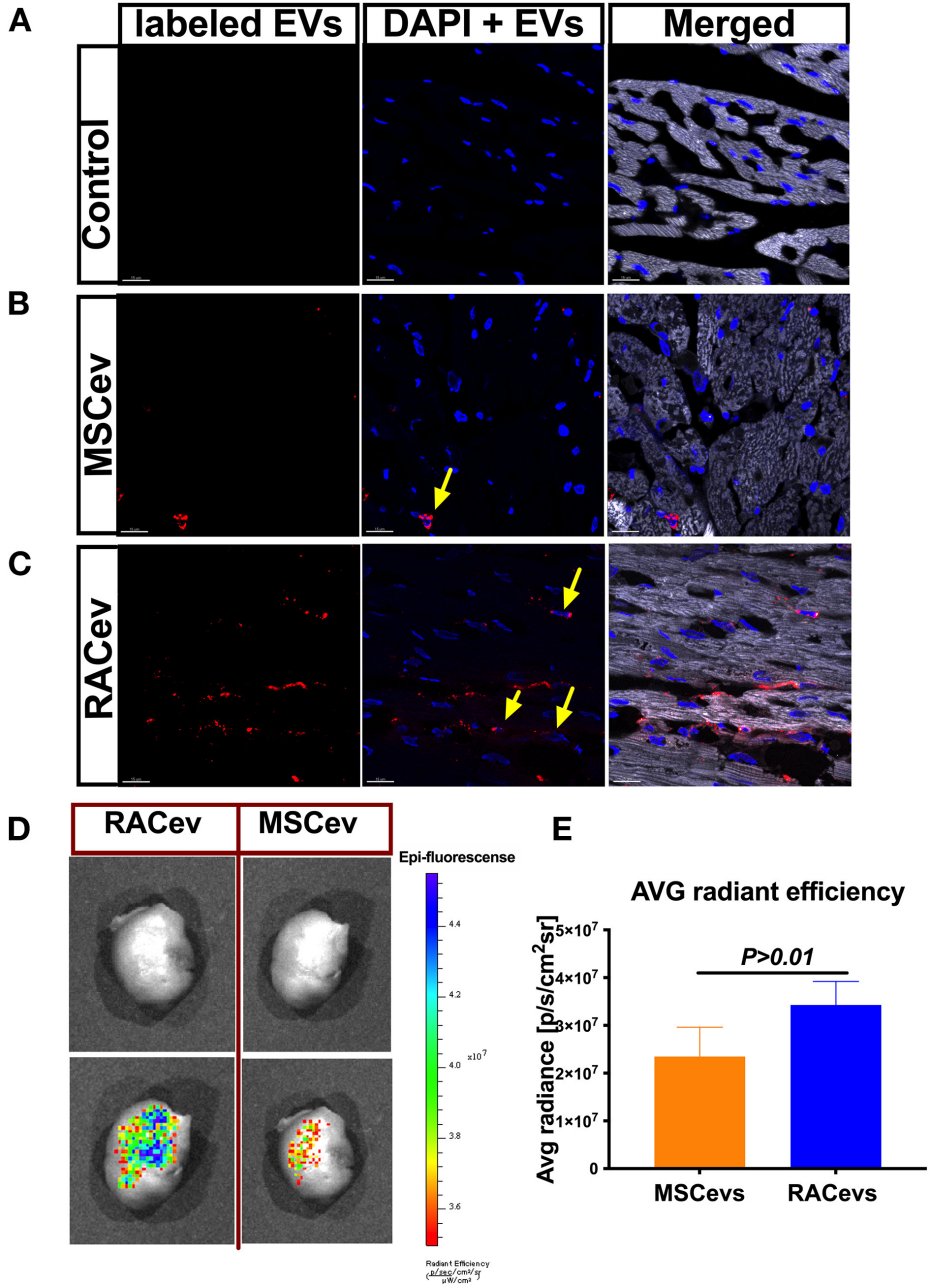
RACev preferentially accumulates to the ischemic myocardium. Representative histological findings of infarcted tissue **(A)** in control groups; **(B)** MSCev showed less infiltration in the ischemic myocardium, whereas **(C)** large amount of RACev accumulated into the infarcted tissue, and the labeled some RACev (red color) were internalized into the cardiomyocyte nuclei (yellow arrows; nuclei stained with Hoechst). **(D,E)** *in vivo* imaging system (IVIS) imaging showed large accumulation of labeled RACev into the infarcted left anterior ventricle. All experiments were repeated twice with the same results. Statistical significance was determined using the Mann-Whitney U test. The results are presented as mean ± SEM (*n* = 6 per group).

We employed an *in vitro* assay to determine the EV internalization time in nucleus-stained (Hoechst) HUVECs. This assay result may indicate the EV internalization time in HUVECs and the *in vivo* experiment termination time for EV tracking in IVIS. According to the *in vitro* findings, the optimal time for the observation of EVs in IVIS was between 3–4 h (Supplementary Figure 2). In immunohistochemistry analysis, RACev beneficially accumulated into the infarcted myocardium, whereas a few MSCev were selectively infiltrated after systemic injection (Figures 6A–C). Furthermore, internalized the transplanted RACev into cardiomyocyte nuclei was higher than MSCev ((Figure 6C). IVIS measurement results demonstrated that the average radiance (p/s/cm^2^sr) was enhanced in the RACev transplanted group compared to the MSCev group (*P* < 0.01, RACev vs. MSCev) (Figures 6D,E), indicating the preferential accumulation of RACev to the site of injury (infarcted anterior left ventricle area) as well as confirming the immunohistology findings.

In summary, EV tracking analyses showed that RACev accumulate selectively in ischemia-induced myocardium.

### Immunotolerance Effects of RACev

**Figure 7:**
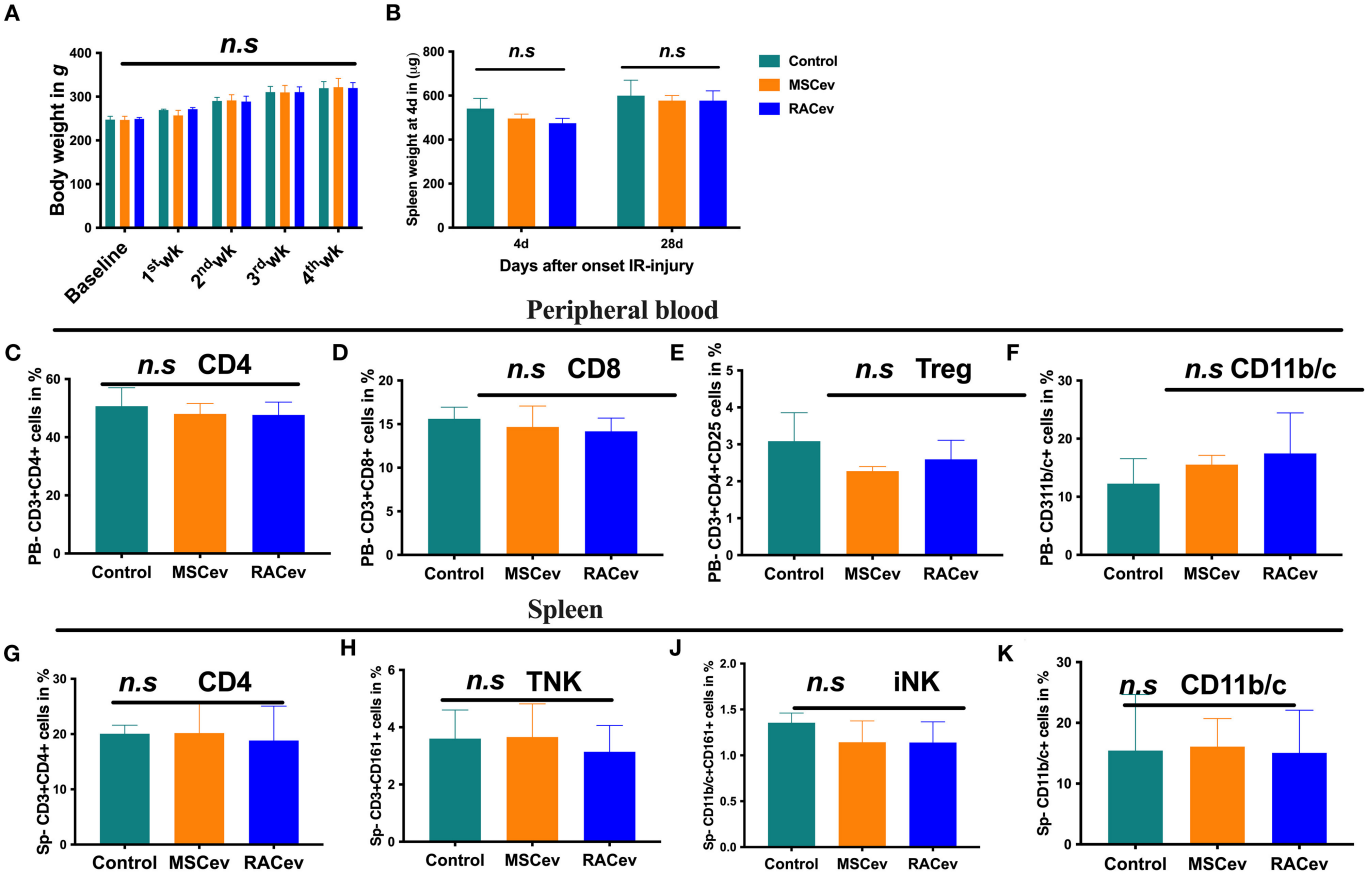
Immunomodulatory effect of RACev. **(A)** The body weights of all three groups were similar across the study period. **(B)** Spleen weight mass at days 4 and 28 days after myocardial ischemia reperfusion injury was not different among the three groups. **(C–F)** Peripheral blood-derived CD4, CD8, Treg, and CD11b/c cells were not elevated, indicating immune tolerance effect of RACev. **(G–K)** Spleen-derived immune reaction or response biomarkers such as CD4, TNK, iNK, and antigen-presenting cells were evaluated and did not change among the three groups. Statistical significance was determined using the One-way ANNOVA followed by Dunn's multiple comparisons *post-hoc* test. The results are presented as mean ± SEM (*n* = 8–10 per group).

A low immunologic response to allogenic EV transplantation is one of the key factors leading to successful organ regeneration. MSCev have already demonstrated superior immunosuppressive effects even in allogeneic settings. Our previous preclinical experiment showed that RACs mainly comprise M2 macrophages, Bregs, and Tregs, and among these, Tregs have a strong immunotolerance effect (12). In this study we evaluated the critical clinical signs of host immunologic reactions for allogenic EV transplantation at day 4 and day 28. As shown in Figure 7A, bodyweight gain and spleen mass were similar in all groups (Figures 7A,B). Moreover, we did not observe any skin ulcer/lesion or alopecia, fur texture changes, or diarrhea in either the RACev-or MSCev-treated groups (data not shown). Further, we performed flow cytometry analysis of the peripheral blood (Figures 7C–F) and spleen (Figures 7G–K) derived CD3+, CD4+, CD8+, Treg, TNK, CD11b/c, and iNK cell numbers which were not elevated and were the same level in all groups. Perhaps transplanted EVs rapidly internalize the damaged tissues (as shown in internalization assay), and peripheral blood immune cells such as antigen-presenting cells may not properly present (or inhibited by miR-142-3p) to the T cells (Supplementary Figures 3A–D). This may indicate less immune reactions occurrence after (even xenogeneic) RACev transplantation at the cellular level.

## Discussion

The therapeutic potential of MSCev for treating ischemic cardiovascular diseases has been well established over the past decade (9). Therefore, in this study, we investigated whether RACev improve the rat myocardial IRI similar to MSCev. In a previous study, Lai et al. transplanted 3.5–1000 μg of proteins derived from MSC exosomes in a mouse or swine model of myocardial ischemia injury, and showed a robust cardioprotective effect along with reduction in the infarct size (9). We first tested the influence of MSCev and RACev on HUVEC proliferation or the cell cycle at different concentrations to determine the optimal dose in an *in vitro* assay. As shown, a high proliferative effect was detected with 5 ^*^ 10^5^ RACev but not with MSCev; this may be linked with MSCev quality and secretion as particle tracking analysis and protein assay indicated that the number of MSCev and protein level is lower (6-fold) than that of RACev. Our transcriptome results provide further insights into the molecular mechanisms underlying the proliferative and anti-apoptotic activities of RACev as they show that miR-181b-3p, miR-150-5p, and miR-302a-5p are significantly upregulated in RACev but not in MSCev. Regarding these, the miR-181 family is reported to promote the endothelial and cancer cell cycle by targeting *CTDSPL* (22, 23). Another study confirmed that miR-150 significantly promoted the migration and tube formation capability of EPCs (24), whereas miR-92a-2-5p acts as an anti-apoptotic miR in endothelial cell lines (24). Thus, the enhanced migration and proliferative effects of RACev may be coupled with these miRs (24, 25). Of these, miR-92a-2-5p enable preservation of ECs and further enhance cell cycle progression (25).

The therapeutic potential of RACs continues to receive widespread attention (12–14, 26, 27). Therefore, for the first time, we focused on the therapeutic implications of RACs derived EVs as a novel paracrine factor secreted by RACs for ischemic diseases. The current study demonstrated that RACev transplantation enhanced heart function significantly, whereas MSCev had a modest effect. The superior effects of RACs may be related to (1) neovascularization, (2) anti-fibrosis (interstitial) and anti-inflammation, (3) cardioprotection and cardiomyogenesis, and (4) preferential accumulation into the ischemic myocardium. The molecular underpinning of paracrine factor-induced neovascularization, anti-fibrosis, and cardiomyogenesis mainly contributes to the therapeutic benefits of myocardial ischemia. Here, we showed that well known regulators of angiogenesis, angiomiR transcripts such as miR-126-3p, miR-126-5p, miR-195-3p, miR-29c-3p, miR-15b-5p, miR-195-5p, miR-200b-5p, miR-146a-3p, and miR-146b-5p are significantly upregulated in RACev than in MSCev, indicating that RACev cargoes constitute key angiomiRs that promote ischemic tissue recovery as shown experimentally (5, 28–32). In a previous study, we demonstrated that vasculogenic EPC numbers are 30-fold higher among RACs than among peripheral blood mononuclear cells, implicating the origin of angiomiRs (12). Moreover, RACev-shuttled angiomiRs as well as anti-fibrosis miRs, which increased capillary density and reduced interstitial fibrosis in immunohistochemical analysis. The miR-133 family is known to control connective tissue growth factor (CTGF), which is a crucial molecule in the process of interstitial fibrosis regulation in rodents as well as in humans (33). Clinical data indicate that the decrease in miR-133 and miR-29c-3p leads to an increase in *CTGF* levels; miR-29c-3p further contributes to collagen synthesis (33). Interestingly, bioinformatics analysis of differentially expressed miRs showed that RACev highly expressed cardiomyogenesis-related miRs such as miR-195, miR-223, miR-208-3p, and miR-499a compared to MSCev. Dueñas et al. (34) showed that miR-195 and miR-223 selectively enhance cardiomyogenesis via *Smurf1* and *Foxp1* driven process. While miR-208 and miR-499 are involved in the late cardiogenic stages mediating differentiation of cardioblasts to cardiomyocytes (35). Furthermore, previous study findings revealed that systemic RACs transplantation increased cardiomyogenesis by upregulation of early cardiac transcriptional cofactors such as *Nkx2-5* and *Gata-4* (12).

Notably, along with their cardioprotective and immunomodulatory effects, MSCs or MSCev are broadly used in allogenic settings as a recommended therapeutic option for cardiovascular diseases due to their lack of HLA class II, such as HLA-DP, HLA-DM, HLA-DQ, and HLA-DR (36, 37). RACs vastly comprise M2Φ, Treg cells, and regulatory B cells (12); of these, Tregs are crucial in allogenic transplantation in terms of strong anti-inflammatory and immunosuppressive properties. Interestingly, RACev transplanted groups showed neither acute (day 4) nor chronic (day 28) host immune response based on clinical signs such as diarrhea, weight loss, and skin lesions (38). The immunomodulatory and immunosuppressive function of RACev may thus be associated with Treg-derived EV secretion, as Tregs modify the host antigen-presenting cell function via miR-142-3p (2285-fold upregulated in RACev), and EVs may escape T cell recognition as shown previously (Supplementary Figure 3E) (39). The miRs have broad functions in immune regulation, for example, miR-10a, miR-21, miR-24 are expressed in immune cells and function as “fine-tuners” for innate and adaptive immune responses (40, 41). In adaptive immunity, they are implicated in the biological processes including pathways involved in the T and B cells central and peripheral tolerance, as well as their function (40). Moreover, flow cytometry findings of PBMCs and spleen cell phenotypes revealed that classical markers of immune reaction such as APC, TNK, iNK, CD8, and CD4/Tregs were similar to the control groups in both the RACev and MSCev groups, indicating the strong immunomodulatory and anti-inflammatory effects of RACev and MSCev miRs.

Emerging evidence highlights that receptor-ligand CXCR4/SDF-1α interaction significantly contributes to the preferential accumulation of EPC-derived EVs into ischemic tissues (42, 43). Mechanistically, RACev may work similarly to the data mentioned above because of their highly selective targeting to the ischemic tissue, as confirmed by IVIS and immunohistology. Moreover, SDF-1α expression in ischemic cardiac tissue is known to be temporarily increased and peaked at 1 to 3 days after ischemic injury and decreased to the background level in the following few days (44–46). Based on this concept, our repetitive EV transplantation timings are suitable and enhance EV uptake by the ischemic myocardium. Moreover, neither RACev nor MSCev changed the concentration numbers when stored at 4°C for up to 1 week; however, when it exceeded 2 weeks, we observed a reduction in the number of EVs (data not shown). Future studies are thus required to determine the optimal storage temperature and biological activity when EVs are stored for longer period using *in vitro* assays.

The majority of previous MSCev based studies used a considerable number of extracellular vesicles for animal studies and obtained striking results. In this study, the limited quantity of MSCev transplantation may not induce similar results as RACev did. There is a limitation that needs to be acknowledged in this study such as allogenic transplantation setting and it‘s safety should be analyzed more precisely.

In conclusion, present findings clearly show that repetitive intravenous infusion of RACev is effective and superior compared to MSCev in terms of cardiac function improvement (ejection fraction, LVDS, and mitral regurgitation), strong neovascularization induction by angiomiRs, anti-fibrosis, anti-inflammatory, and selective accumulation in the ischemic myocardium.

## Data Availability Statement

The data that support the findings of this study are available from the corresponding author upon reasonable request. The data presented in the study are deposited in the NCBI sequence read archive repository, accession number (SUB10448087).

## Ethics Statement

This study was conducted in accordance with the Declaration of Helsinki and was approved by the Tokai University School of Medicine Clinical Investigation Ethics Committee (IRB no. 14R-126 for human samples). All subjects provided written informed consent prior to participating in the study. The patients/participants provided their written informed consent to participate in this study. The animal study was reviewed and approved by the Tokai University School of Medicine Animal Care and Use Committee approved this study (approval # I-20056), based on the Guide for the Care and Use of Laboratory Animals (National Research Council) guidelines.

## Author Contributions

AAS: conception and design, collection and/or assembly of data, data analysis and interpretation, manuscript writing, financial support, and final approval of manuscript. AS and YShe: collection and/or assembly of data, data analysis and interpretation. KY: collection and/or assembly of data and data analysis. YShi: collection and/or assembly of data. SK and TA: conception and design, data analysis and interpretation, manuscript writing, financial support, and final approval of manuscript. All authors contributed to the article and approved the submitted version.

## Funding

This research was supported by JSPS KAKENHI funding (Grant No. 20K17163 to AAS) and Tokai University School of Medicine Research Grant (I-026 to AAS).

## Conflict of Interest

The authors declare that the research was conducted in the absence of any commercial or financial relationships that could be construed as a potential conflict of interest.

## Publisher's Note

All claims expressed in this article are solely those of the authors and do not necessarily represent those of their affiliated organizations, or those of the publisher, the editors and the reviewers. Any product that may be evaluated in this article, or claim that may be made by its manufacturer, is not guaranteed or endorsed by the publisher.

## Supplementary Material

Supplementary Figure 1 RACev enhanced HUVECs proliferation activity in dose-dependently manner. Moreover, cell cycle analysis clearly showed that RACev 250 and 500K treated HUVECs turned into S phase.

Supplementary Figure 2 Internalization assay was performed to define optimal EVs uptaking time by HUVECs. As shown in representative figures that the highest internalization time of labeled EVs to HUVEC was defined at 3 h. Yellow arrows show the labeled EVs (red color) internalized to cell nuclei (blue color is Hoechst-stained nuclei).

Supplementary Figure 3 Host immunologic reactions to xenogeneic EV transplantation was evaluated using peripheral blood and spleen derived T cells subsets. Interestingly, miR-142-3p was significantly upregulated in RACev but not in MSCev. Previous study showed that miR-142-3p has immunotolerance effect by inhibiting antigen presenting cells. Statistical significance was determined using the One-way ANNOVA followed by Dunn's multiple comparisons post-*hoc* test. The results are presented as mean ± SEM (*n* = 6 per group).
