# Supplementary figures and images for: Extracellular Vesicles Derived From Regeneration Associated Cells Preserve Heart Function After Ischemia-Induced Injury
Source: Front Cardiovasc Med. 2021 Oct 20;8:754254. doi: 10.3389/fcvm.2021.754254 (PMC8564358; doi:10.3389/fcvm.2021.754254)

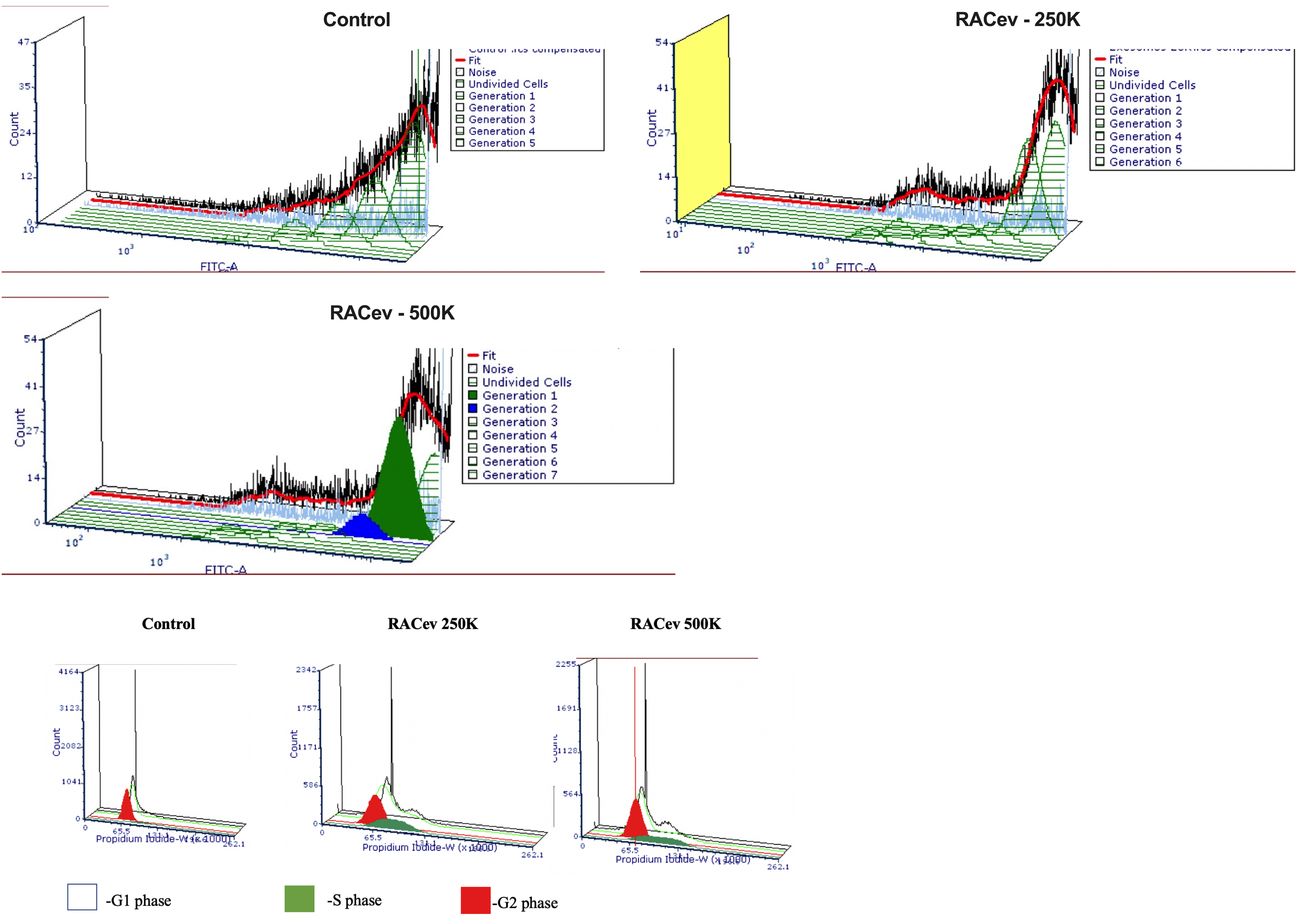

Supplement: Supplementary Figure 1 — RACev enhanced HUVECs proliferation activity in dose-dependently manner. Moreover, cell cycle analysis clearly showed that RACev 250 and 500K treated HUVECs turned into S phase. [file Image_1.TIFF]

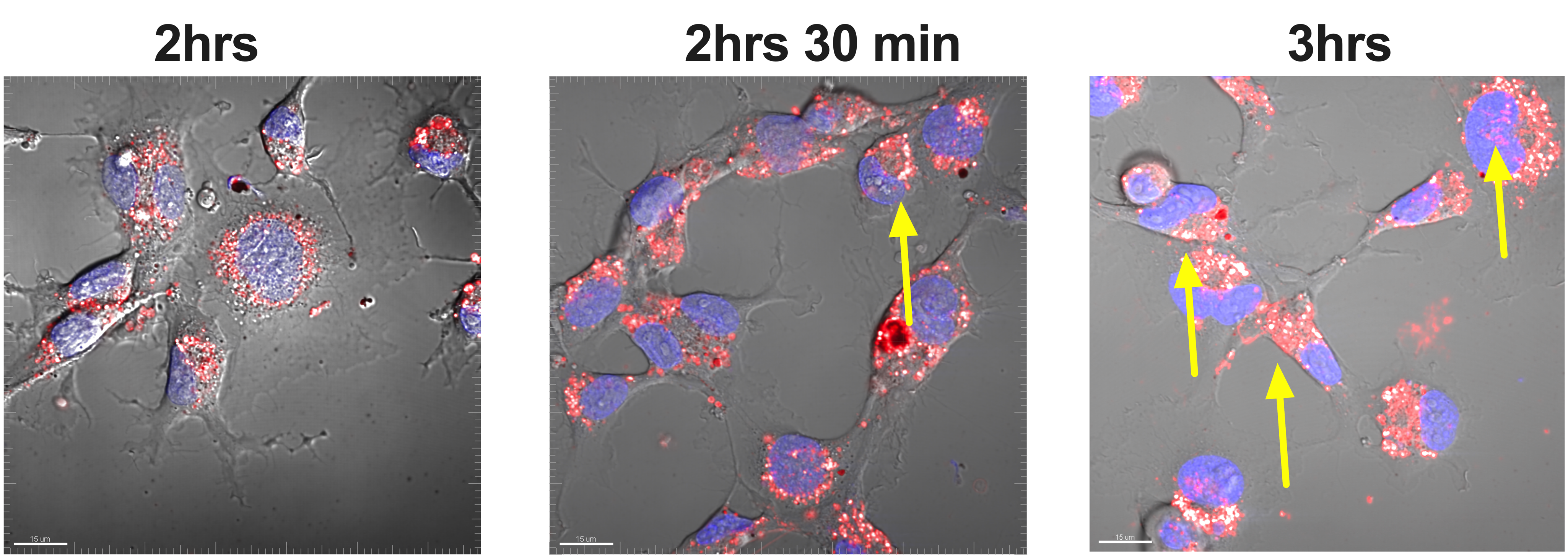

Supplement: Supplementary Figure 2 — Internalization assay was performed to define optimal EVs uptaking time by HUVECs. As shown in representative figures that the highest internalization time of labeled EVs to HUVEC was defined at 3 h. Yellow arrows show the labeled EVs (red color) internalized to cell nuclei (blue color is Hoechst-stained nuclei). [file Image_2.TIFF]

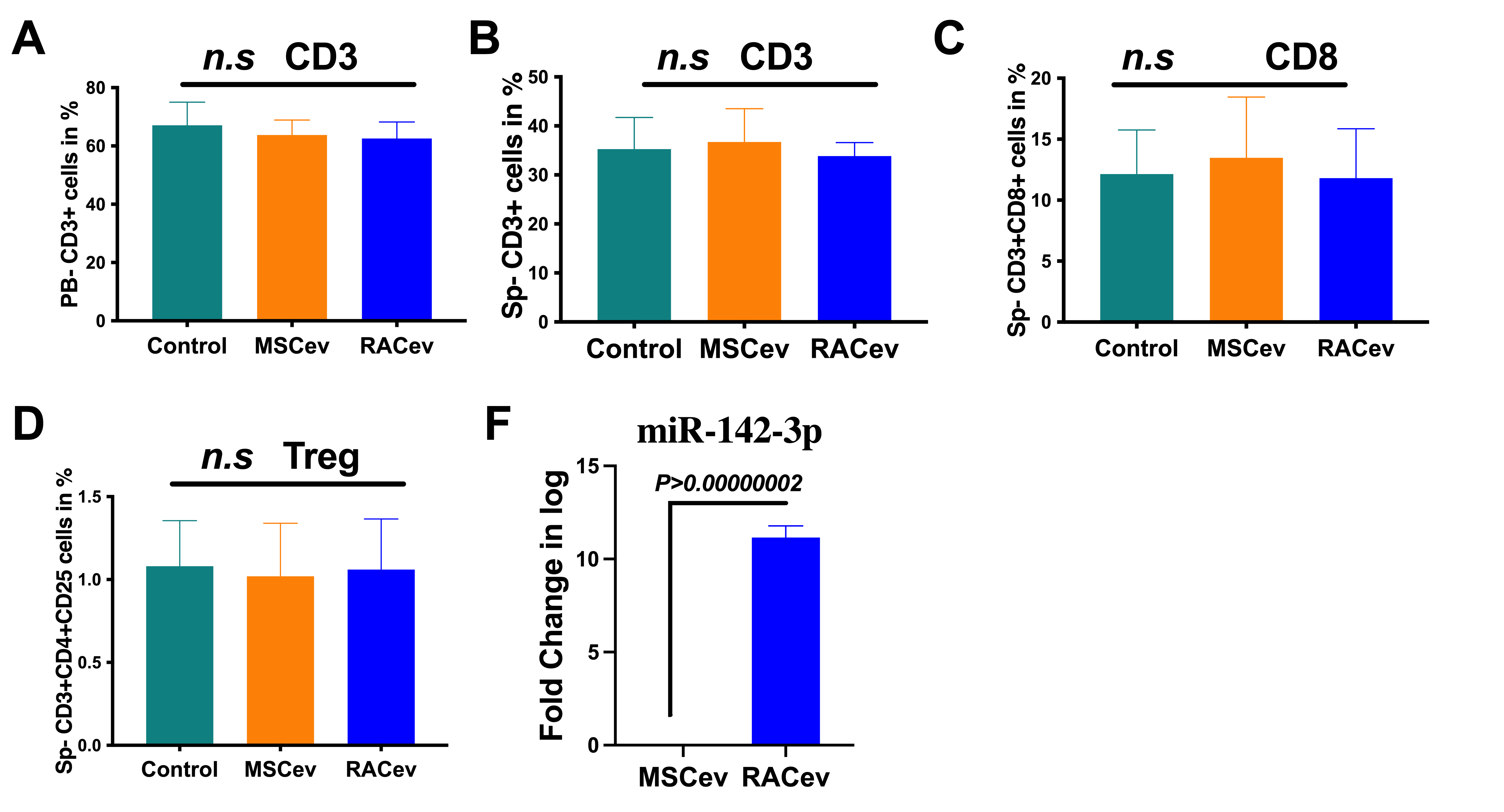

Supplement: Supplementary Figure 3 — Host immunologic reactions to xenogeneic EV transplantation was evaluated using peripheral blood and spleen derived T cells subsets. Interestingly, miR-142-3p was significantly upregulated in RACev but not in MSCev. Previous study showed that miR-142-3p has immunotolerance effect by inhibiting antigen presenting cells. Statistical significance was determined using the One-way ANNOVA followed by Dunn's multiple comparisons post-hoc test. The results are presented as mean ± SEM (n = 6 per group). [file Image_3.TIFF]
